# Label-free, fast, 2-photon volume imaging of the organization of neurons and glia in the enteric nervous system

**DOI:** 10.3389/fnana.2022.1070062

**Published:** 2023-02-09

**Authors:** Doriane Hazart, Brigitte Delhomme, Martin Oheim, Clément Ricard

**Affiliations:** Université Paris Cité, CNRS, Saints-Pères Paris Institute for the Neurosciences, Paris, France

**Keywords:** enteric nervous system (ENS), two-photon microscopy, autofluorescence, Meissner’s plexus, Auerbach’s plexus, tissue clearing

## Abstract

The enteric nervous system (ENS), sometimes referred to as a “second brain” is a quasi-autonomous nervous system, made up of interconnected plexuses organized in a mesh-like network lining the gastrointestinal tract. Originally described as an actor in the regulation of digestion, bowel contraction, and intestinal secretion, the implications of the ENS in various central neuropathologies has recently been demonstrated. However, with a few exceptions, the morphology and pathologic alterations of the ENS have mostly been studied on thin sections of the intestinal wall or, alternatively, in dissected explants. Precious information on the three-dimensional (3-D) architecture and connectivity is hence lost. Here, we propose the fast, label-free 3-D imaging of the ENS, based on intrinsic signals. We used a custom, fast tissue-clearing protocol based on a high refractive-index aqueous solution to increase the imaging depth and allow us the detection of faint signals and we characterized the autofluorescence (AF) from the various cellular and sub-cellular components of the ENS. Validation by immunofluorescence and spectral recordings complete this groundwork. Then, we demonstrate the rapid acquisition of detailed 3-D image stacks from unlabeled mouse ileum and colon, across the whole intestinal wall and including both the myenteric and submucosal enteric nervous plexuses using a new spinning-disk two-photon (2P) microscope. The combination of fast clearing (less than 15 min for 73% transparency), AF detection and rapid volume imaging [less than 1 min for the acquisition of a z-stack of 100 planes (150*150 μm) at sub-300-nm spatial resolution] opens up the possibility for new applications in fundamental and clinical research.

## Graphical Abstract

Combined fast clearing and two-photon volume autofluorescence imaging allow for label-free anatomical studies of intestinal tissue and biopsies at unprecedented speed and detail. 
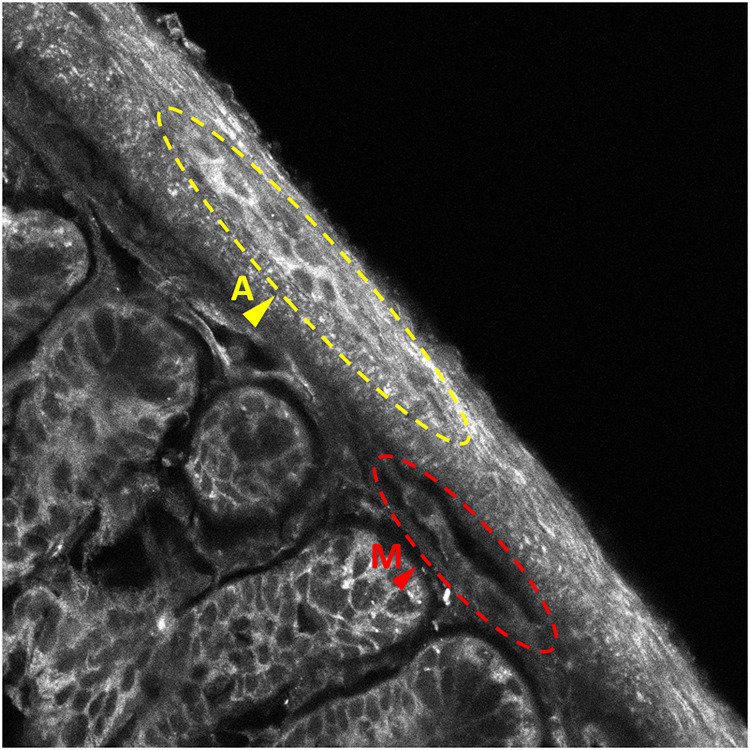

## 1. Introduction

The enteric nervous system (ENS) has been referred to as a “second brain” for many decades (Gershon, 1998; Avetisyan et al., 2015; Schneider et al., 2019). Comprising more than 100 million neurons organized in a meshwork of interconnected ganglia, the ENS is orchestrating all intestinal functions including bowel motility, mucosal secretion, modulation of endocrine secretion, blood-flow regulation, and immune response. Although largely autonomous in its function, the ENS has extensive connections with both the sympathetic and parasympathetic systems, engaging a bi-directional signaling with the CNS through the vagal nerves [reviewed in Costa et al. (2000), Avetisyan et al. (2015), and Schneider et al. (2019)].

### 1.1. Imaging enteric neural networks

**FIGURE 1:**
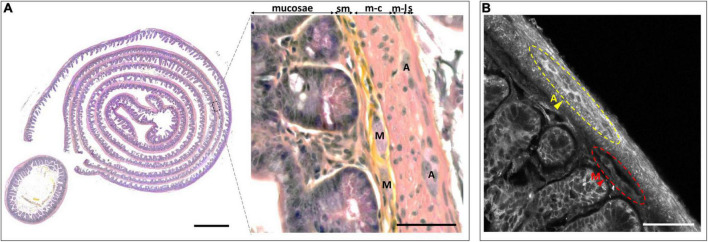
Benchmark histological observation of the enteric nervous system (ENS). **(A)** Hematoxylin/erythrosine/saffron (HES) stained intestinal Swiss roll (left) and transversal slice (left-inset). Magnification of a part of the intestinal wall observed on the Swiss roll, highlighting the different layers and components of the ENS (right). sm, *submucosa*; m-c, *muscularis circular*; m-l, *muscularis longitudinalis*; s, *serosa*; A, Auerbach’s *plexus*; M, Meissner’s *plexus*; Scale-bars: 500 μm (left), 50 μm (right). **(B)** Autofluorescence (AF) imaging from an intestinal Swiss roll under 405-nm excitation. A, Auerbach’s *plexus*; M, Meissner’s *plexus*; Scale-bar: 50 μm.

Traditional studies of the anatomy and pathology of the ENS have relied either on whole-mount tissue staining (Lebouvier et al., 2010b) or on slicing, often combined with hematoxylin and eosin (H and E) staining and followed by imaging of selected single thin sections with bright-field microscopy. This approach makes it unlikely to capture the intricate three-dimensional (3-D) architecture of the ENS characterized by two interconnected types of ganglia in the intestinal wall. The myenteric *plexus* (also known as *plexus* of Auerbach) is located in between the circular and longitudinal *muscularis*, two orthogonal layers of smooth muscle cells that are responsible for the motility of the bowel. Neurons of the myenteric *plexus* mainly regulate the motor function of the gut. The submucosal *plexus* is located in vicinity of the gut *lumen*. It mainly regulates secretory functions (Kulkarni et al., 2018; Schneider et al., 2019; Figure 1). Both Auerbach’s and Meissner’s *plexus* are composed of different neuronal subtypes as well as enteric glia that interact with one another and they target different effectors (Boesmans et al., 2013; Schneider et al., 2019). This layered organization is best revealed on transverse intestinal sections. On the other hand, longitudinal sections better reveal the interconnections between these ganglia, which line the intestine like a fishnet (Costa et al., 2000). Imaging this 3-D structure comprising the dispersed neurites, fibers, and structural components is challenging, but it is of considerable interest.

Abnormalities of the ENS have been linked to many pathologies of the digestive tract. One drastic example is Hirschsprung’s disease where regions of the large intestine (colon) lack ganglia. Without these nerve cells stimulating gut muscles to move contents through the colon, the contents can back up and cause constipation. More surprisingly, ENS abnormalities are linked to central neuropathologies, too. For example, the analysis of the ENS by routine colonoscopy biopsies was shown to offer a pre-mortem diagnostic tool of Parkinson’s disease (PD), and studying ENS alterations provides insight into the progression of motor and non-motor symptoms, too (Lebouvier et al., 2010b; Ohlsson and Englund, 2019; Schneider et al., 2019). In addition, ENS dysfunction has been linked to other central pathologies, like autism (Israelyan and Margolis, 2018; Heiss and Olofsson, 2019), underpinning the need and interest of a detailed morphological characterization of alterations in its architecture with modern imaging techniques.

Detailed 3-D observations have been rare and they mainly comprise very limited tissue volumes (see, however, Cirillo et al., 2013; Fung et al., 2021). Typical 3-D reconstructions use serial thin sections, immunolabeling and their sequential observation with confocal microscopy, followed by image processing and 3-D rendering. Also, immunofluorescence protocols already require many hours on thin slices and days for the incubation of such thick samples. An additional bottleneck comes from the speed-limit of confocal laser-scanning microscopy (CLSM), which is inherently slow for reconstructing such large volumes, because the image is acquired and built up point-wise. In addition, as the entire sample volume is illuminated but fluorescence only collected only from a single spot, confocal imaging rapidly photobleaches the entire volume during volume scans. Thus, this serial two-dimensional (2-D) approach is extremely time-consuming for larger samples like the ENS that measures 3 mm for a transverse section of mouse colon and more for biopsies.

Two-photon (2P) excitation fluorescence microscopy (Denk et al., 1990) has become the gold standard for imaging thick tissue samples (Denk and Svoboda, 1997) because the better penetration of IR excitation and collection of scattered fluorescence (Oheim et al., 2001). Hundreds of fluorophores have action cross-sections that make them amenable to 2P imaging (Ricard et al., 2018) and even tissue autofluorescence (AF) can be used to generate intrinsic contrast and provide context information [see, e.g., Zipfel et al., 2003]. 2P microscopy and AF detection have been used for imaging various parts of the gut, including the stomach (Rogart et al., 2008), the small intestine (Orzekowsky-Schroeder et al., 2011; Ricard et al., 2012), and the colon (Aggarwal et al., 2013). Yet, 2P scanning–as CLSM–scales with the number of tissue voxels imaged and still requires 6–8 s/frame (Ricard et al., 2012) and several minutes for imaging a single block of intestinal tissue.

### 1.2. Fast, multi-spot, multi-photon imaging of rapidly cleared samples

Multi-spot 2P-imaging techniques parallelize either fluorescence excitation and/or pixel readout to overcome the low frame rate of single-spot scanning schemes (Bewersdorf et al., 2006). The most common of these are 2-P multi-spot scanning systems (Kim et al., 1999; Nielsen et al., 2001) and 2-P spinning-disks microscopes (Egner and Hell, 2000; Straub et al., 2000; Kobayashi et al., 2002). Both geometries have in common that they associate a planar non-linear fluorescence excitation with a “one-shot” readout on a fast camera detector. By design, these systems compromise on optical sectioning and image contrast, as cross talk between neighboring pinholes and image blurring by scattered fluorescence becomes an issue, even at moderate imaging depths [see, e.g., Shimozawa et al. (2013) for discussion].

An alternative consists in generating a thin sheet of light orthogonal to the microscope axis, either by cylindrical optics and/or scanning the beam in a plane. The excited fluorescence is collected perpendicular to the illuminated plane in a separate collection optical path and imaged onto a large-format camera. This configuration combines improved contrast and axial sectioning with short acquisition and it reduces phototoxicity because only the imaged plane is illuminated at a given time (Huisken et al., 2004). However, even in its 2-P excitation variant (Truong et al., 2011; Mahou et al., 2014; Zong et al., 2015), light-sheet microscopes suffer from a reduced *z*-resolution compared to spot-scanning systems.

In response to these challenges, we recently introduced a 2P microscope produces a planar non-linear excitation but that retains the classical inline geometry with a single objective lens (Rakotoson et al., 2019). Its optical sectioning, high frame rate and low photograph damage are achieved through spatially and temporally multiplexing 2P excitation for planar illumination. When using a high-numerical aperture (NA) low-magnification objective lens (Oheim et al., 2001) some 50 excitation spots are scanned into the focal plane, with minimally 12 ms needed for a homogenously lit full-frame acquisition (and more for dimmer samples). Our instrument combines the speed, field-of-view and ease of a 2P light-sheet microscope with the resolution and image contrast of a scanning 2P microscope. We previously demonstrated simultaneous dual-color 3-D imaging of brain organoids derived from human inducible pluripotent stem cells (hIPSCs) with 300 nm lateral and 1.5 μm axial resolution over large fields of view. We reached an imaging depth exceeding 200 μm at a speed 10–100-times faster than a single-spot scanning microscope (Rakotoson et al., 2019). Under realistic (low mW) excitation powers and signal levels typical for AF imaging or the detection of faint biological labels, two 900 × 900 pixel dual-color images were simultaneously captured in 480 ms. These characteristics make our microscope attractive for 3-D imaging of healthy and pathological thick intestinal samples.

One major drawback common to all parallelized 2-P imaging systems is that they work best with transparent samples or in superficial tissue layers. The reason is that a major advantage of single-spot 2-P imaging is sacrificed by using an imaging detector rather than a “bucket” detector just collecting photons-the possibility of making efficient use of multiply scattered fluorescence (Oheim et al., 2001). Therefore, recent efforts in improving and speeding up volume imaging have typically gone along with improvements in tissue clearing [reviewed in Richardson and Lichtman (2015), Ueda et al. (2020), Tian et al. (2021), and Weiss et al. (2021)].

A plethora of different optical clearing approaches have been proposed to meet the demand for 3-D volume imaging [see, e.g., Neckel et al. (2016), Bossolani et al. (2019), Yuan et al. (2021) for work in the ENS]. Each technique has its own strengths but still suffers from either low transparency, long incubation time, processing complexity, tissue deformation, or fluorescence quenching (or a combination of these), and a single solution that best satisfies all aspects has been missing. In the present work, we combine a new patent-pending fast, universal tissue-clearing (Delhomme et al., in preparation) and volumetric-imaging strategies for imaging the mouse small intestine. We demonstrate a highly contrasted 3-D reconstruction of enteric ganglia entirely based on the AF-based identification of neurons and glial cells of the ENS. Our manuscript is organized as follows: (*i*) we first describe the fast clearing approach of intestine sample compatible with the conservation of AF signals; (*ii*) characterizing AF spectra and intensities at a cellular and sub-cellular scale, we reliably identify on label-free images neurons and enteric glial cells in both Auerbach’s and Meissner’s plexus; (*iii*) we validate this AF-based identification of by confocal immunofluorescence. Finally, (*iv*) using our fast 2P spinning-disk microscope, we demonstrate volumetric label-free imaging of the ENS throughout the entire depth of the intestinal wall. We expect the here proposed workflow associating fast clearing and rapid 3-D imaging to be valuable for future biomedical applications, e.g., during diagnostic imaging.

## 2. Materials and methods

### 2.1. Ethics statement

All experiments were performed according to the French legislation and in compliance with the European Community Council Directive of November 24, 1986 (86/609/EEC) for the care and use of laboratory animals. The used protocols were approved by the local Ethics Committee. In total, this study used 12 C57BL/6 mice (Charles River) that were bred and housed in the local animal house on an inverted dark-light cycle with access to food and water *ad libitum*.

### 2.2. Sample preparation

#### 2.2.1. Intestine extraction and fixation

Mice were killed with CO_2_ and their small intestine was dissected out, flushed and washed with a solution of 1X Phosphate-Buffered Saline (1X PBS) at pH 7.2 (Gibco, Waltham, MA, USA) and cut into three parts: *duodenum*, *jejunum*, and *ileum*, which were fixed overnight (ON) at 4°C in formalin (VWR, Fontenay-sous-Bois, France), a 4% formaldehyde solution buffered to pH 6.9. The thus cleaned samples were stored until use in 1X PBS/0.02% NaN_3_ at 4°C. All of the experiments done on small intestines were realized on *ileum* sections. The same protocol was used for the experiments done on colon.

#### 2.2.2. Freezing and cryostat section of samples

Fixed tissue was cut into 1-cm long fragments and first impregnated during 3 h in a 15%-sucrose solution (Merck, Darmstadt, Germany) in 1X PBS, 0.02% NaN_3_. Samples were placed in a similar solution, but containing 30%-sucrose solution for cryoprotection before being embedded with optimal cutting temperature (OCT, VWR, Rosny-sous-Bois, France) (CellPath) and immersed in ice-cold isopentane in dry ice. Once frozen, 7 μm thin transversal slices were cut on a cryostat (Reicher-Jung, Cryocut 1800; Leica, Wetzlar, Germany) and deposited on positively charged BK-7 glass slides (Menzel-Gläser, Braunschweig, Germany, Superfrost Plus; Gerhard Menzel). The thus prepared slides were conserved at −80°C until use.

#### 2.2.3. Swiss roll

Fixed mouse intestinal tissues were incised longitudinally to expose the lumen (Bialkowska et al., 2016). The sample was processed as described above and—after the cryoprotective step—using a wooden toothpick—the samples were rolled up on themselves, the luminal side facing the inside. The thus obtained rolls were placed in plastic molds filled with OCT and frozen at −80°C. Finally, 7 μm thick cryostat sections were cut, giving the characteristic swill-roll shape.

### 2.3. Staining, immunofluorescent labeling, and clearing

Due to the long incubation times in each protocol, all reagents used contained 0.02% NaN_3_ as a preservative. Room temperature (RT) was 20–25°C.

#### 2.3.1. Hematoxylin/Erythrosine/Saffron (HES) staining

Intestines were washed in PBS and fixed overnight in a 4% formaldehyde solution (Merck, Darmstadt, Germany). Then, they were stored in ethanol 70% before embedding. Tissues were dehydrated in solutions with increasing concentrations of ethanol, then isopropanol, and finally impregnated in paraffin (Logos One, Thermo Fischer, Waltham, MA, USA), before embedding. Slides were deparaffinized and stained for HES (Hematoxylin/Erythrosine/Saffron): in this protocol, nuclei are stained with hematoxylin in blue, cytoplasm in pink with erythrosine and collagen fibers in yellow by saffron. Slides were stained and dehydrated in automaton (SPECTRA, Leica, Wetzlar, Germany), then mounted in a permanent mounting medium (Pertex).

#### 2.3.2. Thick-sample immunolabeling

The previously fixed tissues were cut into 1.5 cm long sections. An antigen retrieval step by successive methanol baths ensuring a dehydration/delipidation of the cells was performed at RT, followed by a permeabilization step with an aqueous solution of 0.2% Triton X100 (Bio-Rad Laboratories, Marnes-la-Coquette, France), 0.2 M Tris pH8, 20% DMSO and 0.3 M Glycine for 1 h, followed by a blocking step using an aqueous solution of 0.2% Triton X100 (Bio-Rad Laboratories, Marnes-la-Coquette, France. Marnes-la-Coquette, France), 0.2 M Tris pH8, 10% DMSO and 10% goat serum for 5 h, all under agitation at 37°C. Tissue slices were incubated with a primary antibody anti-HuC/D (1/250; Ab184267; Abcam, Paris, France) or anti-S100β (1/100; ab52642; Abcam, Paris, France) in an aqueous solution of 10% washing buffer 10X, 10% goat serum and 5% DMSO in Eppendorf tube for 40 h under agitation at 37°C. After five successive washes of 30 min and another wash of 1 h under agitation at RT with an aqueous solution of 0.2 M Tris pH8 and 0.2% Tween 20, the samples were incubated with a secondary antibody (Goat anti rabbit conjugated Alexa fluor 633; 1/1000; A-21070; Invitrogen, Waltham, MA, USA) in an aqueous solution containing 10% washing buffer 10X, 10% goat serum and 5% DMSO for 20 h, under agitation, in the dark at 37°C. The samples were then washed as before and were stored protected from light prior to imaging.

#### 2.3.3. Thick-sample clearing

Optical tissue clearing is the result of delipidation and RI (refractive index) homogenization (Richardson and Lichtman, 2015). The exact formulation of the here used reagents cannot be released at this time, DPG and BRG are being patented (INPI deposit n° P7435FR00-51656). A publication describing the workflow in detail is in underway (Delhomme et al., in preparation). Briefly, DPG, the depigmentation solution, contains hydrogen peroxide. BRG contains a mixture of detergent, buffer, and 60% 2,2’ Thiodiethanol (TDE) (2,2′-thiodiethanol). The RI of the mixture is 1.47. Clearing of the full small intestine sample is achieved in less than 20 min at 37°C, in one step, without the requirement of shuttling the sample through various solutions of different concentration.

### 2.4. Imaging

#### 2.4.1. Brightfield

Images of the HES staining were taken on an Axioscan Z1 (Zeiss, Oberkochen, Germany) equipped with a × 40/NA0.95 air objective.

#### 2.4.2. Macroscope

Images illustrating the clearing steps of thick intestinal sample were obtained with the SMZ800 stereo macroscope, equipped with a digital camera DS-Fi and DS-U2/L2 USB (Nikon, Amstelveen, The Netherlands). Samples were illuminated with white light (3,000°K) and the images acquired with the NIS-Element software.

#### 2.4.3. Confocal microscopy

Images of tissue sections and thick samples were acquired on either a LSM880 inverted confocal Airyscan or LSM710 META upright laser scanning microscope (both from Zeiss, Oberkochen, Germany). AF images were obtained following 405 nm excitation, immunolabeling images under 633 nm excitation. The objectives used were a ×40/NA1.2 water-immersion objective (Zeiss Plan-Apochromat, Oberkochen, Germany), a x25/NA0.8 multi-immersion (Zeiss Plan-Apochromat), and a x20/NA0.8 (Zeiss Plan-Apochromat). All images were acquired with the pinhole diameter set to 1 Airy unit. AF was collected in between 417 and 740 nm. For immunolabeling acquisitions signal was collected in between 645 and 735 nm and AF in between 415 and 599 nm.

#### 2.4.4. Non-linear microscopy

The OASIS 2P spinning-disk microscope is described elsewhere (Rakotoson et al., 2019). Briefly, 2P images were acquired upon 800-nm excitation and the signal was collected in between 400 and 568 nm. Acquisition time was 480 ms for each AF image.

### 2.5. Image analysis, quantification, and statistics

#### 2.5.1. Image processing

Images were visualized and analyzed with the IMAGEJ/FIJI (Schindelin et al., 2012). 3-D rendering and animation were performed in IMARIS (Bitplane-Oxford Instruments, Zürich, Switzerland). Data were processed with EXCEL (Microsoft, Redmond, WA) and PRISM (GraphPad, San Francisco, CA) software.

#### 2.5.2. Clearing analysis

1.5 cm long intestinal sections were split into two groups. One group (*n* = 6) was depigmented during 45 min with an in-house DPG solution (patent pending); the other (*n* = 4) was used as a control and not depigmented. Either samples were placed in a home-made tank with a black-and-white stripe pattern below its glass bottom. Images were acquired on a SMZ800 stereo macroscope (Nikon, Amstelveen, The Netherlands) as described above. A first image was acquired with the sample immersed in a 0.2 M Tris solution as a reference. The Tris solution was then removed and replaced with our BRG5 clearing solution (patent pending). Images were acquired every 10 s during 5 min, then every 30 s during 5 min and finally every minute during 10 min. For each image, we calculated Michelson’s contrast *C*_*M*_ = (*I*_*max*_−*I*_*min*_)/(*I*_*max*_ + *I*_*min*_) using the intensity measured on the black (*I*_*min*_) and white stripes (*I*_*max*_). Data were processed with PRISM (GraphPad, San Francisco, CA, USA) software and the half-life, *t*_1/2_, and plateau value of the evolution of *C*_*M*_ with time extracted for either condition (depigmented vs. non-depigmented).

#### 2.5.3. Spectral analysis

Autofluorescence spectra were acquired upon 405 nm excitation from non-depigmented cleared intestinal samples. Spectral images were taken at the level of Auerbach’s and Meissner’s plexus, using the 32-element spectral PMT array of a LSM880 confocal microscope (Zeiss). At the level of the Auerbach’s plexus, regions of interest (ROIs) were selected in the cytoplasm and nuclei of neurons, in the cytoplasm of enteric glia and in the *muscularis*. At the level of Meissner’s plexus, ROIs were selected in the cytoplasm and nuclei of neurons and in the *submucosa*. Mean fluorescence intensities were measured and normalized using the ImageJ software. The shown data are from eight and six independent acquisitions at the level of Auerbach’s and Meissner’s plexus, respectively.

#### 2.5.4. Statistics

All results are at least triplicates of independent experiments and are represented as mean ± SD. Mann–Whitney test was used to compare among experiments.

## 3. Results

### 3.1. Conventional histological observation is ill-suited for studying the 3-D organization and connectivity of the ENS

In clinical neurogastroenterology, as well as in fundamental research, the ENS is mainly being studied using histological techniques that involve serial slicing, staining, and observation steps. As a consequence, on a typical transverse slice of the mouse intestine, it is very rare to find both Auerbach’s and Meissner’s plexus, and the connections in between them are lost (Figure 1A–left panel, inset).

An alternative is the “Swiss roll,” which increases the probability of finding both plexuses (Bialkowska et al., 2016; Figure 1A–left). The probability of finding both nervous plexuses within the same field of view increases dramatically (Figure 1A–zoom), however, despite its advantages, it is neither possible to observe the full 3-D architecture of the ENS, nor to study its connections with peripheral and central nerves.

Notwithstanding these limitations, we prepared a Swiss-roll ENS section and stained it with HES (Figure 1A–right panel)–an approach that can only be applied to thin slices–to provide a benchmark image against which we will have to hold our new method. On the other hand, in preliminary experiments, we observed a distinct tissue AF that produced sufficient contrast for identifying both plexuses (Figure 1B). We therefore set out to study if this label-free technique, with appropriate 3-D imaging techniques, would be amenable to a full volumetric reconstruction of intact (i.e., non-sliced) intestinal samples.

### 3.2. A fast clearing protocol compatible with autofluorescence imaging

In intact intestinal samples the imaging depth is limited by the scattering of both excitation light and the generated fluorescence by tissue components—less by absorption that occurs mainly through pigments. Clearing techniques enhance tissue transparency and/or homogenize its RI [see, e.g., Avilov (2021), Richardson et al. (2021), Tian et al. (2021), and Weiss et al. (2021) for recent reviews]. Together with advances in 3-D microscopy, clearing techniques have permitted detailed volumetric reconstructions of large tissue volumes, also of the intestine (Liu et al., 2011; Bossolani et al., 2019; Graham et al., 2020).

**FIGURE 2:**
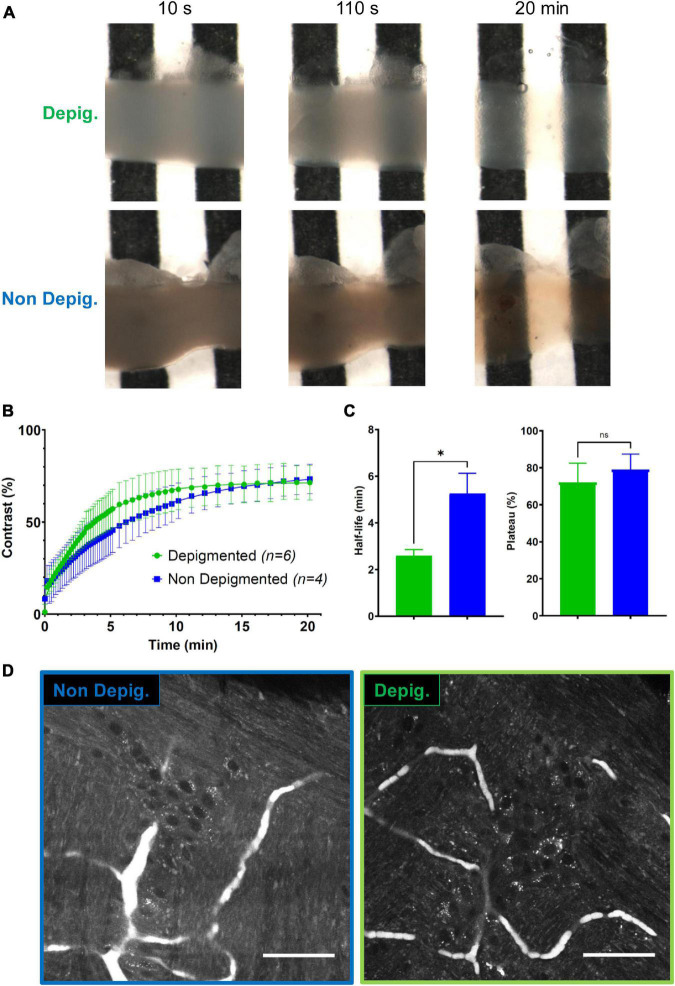
Fast clearing of the small intestine. **(A)** Longitudinal sections of *ileum* observed at various delays after the addition the clearing reagent (10 s, 110 s, 20 min) on a black and white pattern with (top) or without (bottom) a depigmentation step. **(B)** Quantification of the constrast (*C*_M_) enhancement under depigmented (green) and non-depigmented (blue) conditions. **(C)** Half-life (left) and yield (efficiency) of the clearing protocol (right) under depigmented (green) and non-depigmented (blue) conditions. **(D)** Autofluorescence (AF) imaging at the level of the Auerbach’s *plexus* upon 405 nm excitation in non-depigmented (left) and depigmented (right) conditions. Scale-bar: 50 μm. Error-bars show SD. *, *p* < 0.05, Mann–Whitney test; ns, non-significant.

To significantly speed up the workflow of volumetric tissue imaging, we developed BRG5, an ultra-fast, universal, non-toxic clearing protocol, based on a high-RI aqueous solution that was successfully tested on various organs (Delhomme et al., in preparation). Intrinsic imaging requires the clearing to have a minimally attenuating effect on the already faint AF signal. We compared two protocols, one of which involving a depigmentation step of 45 min. Using intact intestinal samples of 3 × 3 × 10 mm, we compared the efficacy of the clearing on depigmented and non-depigmented tissue. In either case, the whole intestine became rapidly transparent upon treatment with BRG5, and the depigmentation had little if any effect on the final transparency (Figure 2A). We substantiated this observation by the quantification of the evolution with time of Michelson’s contrast *C*_*M*_ (visibility) of a black and white stripe pattern placed underneath intestinal samples (Figure 2B). In either condition, a plateau was reached in less than 15 min and the final contrast attained was undistinguishable (71 vs. 73%; Figure 2C–right panel). The only parameter that was significantly different was the half-time for clearing (2 min 36 s for depigmented vs. 5 min 18 s for non-depigmented samples; Figure 2C–left panel). However, this advantage seems negligible in view of the extra 45 min added when using the depigmentation process. In addition, AF images of Auerbach’s plexus acquired upon 405-nm excitation revealed a stronger signal and higher contrast without depigmentation (Figure 2D). We conclude that depigmentation of the intestine is dispensable and that clearing of the whole intestinal wall can be performed in less than 15 min without destroying the AF signal.

### 3.3. Autofluorescence imaging allows a detailed morphological characterization of the ENS

**FIGURE 3:**
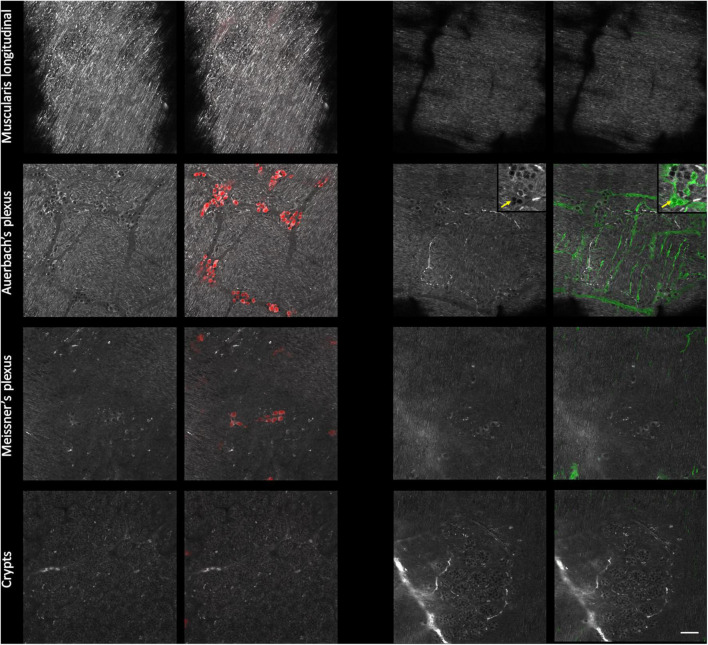
Immunohistological characterization of the autofluorescent structures of the enteric nervous system (ENS). Single autofluorescent images (grayscale) taken at different depth in the intestinal wall (*muscularis longitudinalis*, Auerbach’s *plexus*, Meissner’s *plexus*, and crypts) and corresponding immunolabeled images. Red: neurons cell bodies (Hu C/D); green: enteric glia (S100β); *inset*: crop showing enteric glia and AF colocalization with S100β immunolabeling; yellow arrow: enteric glia cell bodies. Scale-bar: 50 μm.

We next confirmed that the AF structures were indeed neural cells by immunolabeling against HuC/D, a pan-neuronal marker (Mazzoni et al., 2021) and against S100β, an enteric-glia marker (Grundmann et al., 2019). As expected, we detected no specific labeling at the level of the *muscularis longitudinalis* (Figure 3–first line). Within Auerbach’s plexus (Figure 3–second row), neuronal cell bodies were located in the structures previously identified as ganglia on the AF images. Neurons exhibited strongly AF granules in their cell bodies. The colocalization between AF signals showing small cell bodies without granules and S100β immunolabeling allowed us to unambiguously pinpoint enteric glia on AF images of Auerbach’s plexus. Likewise, HuC/D reliably labeled neurons in the ganglia identified as Meissner’s plexus (Figure 3–third row), albeit at lower density compared to Auerbach’s plexus. Also, neurons in Meissner’s plexus exhibited less AF granules than those found in Auerbach’s plexus, and enteric glia were not detectable at this level. Finally, crypts exhibited solely AF signal (Figure 3–bottom row).

### 3.4. Spectral characterization of ENS autofluorescence

**FIGURE 4:**
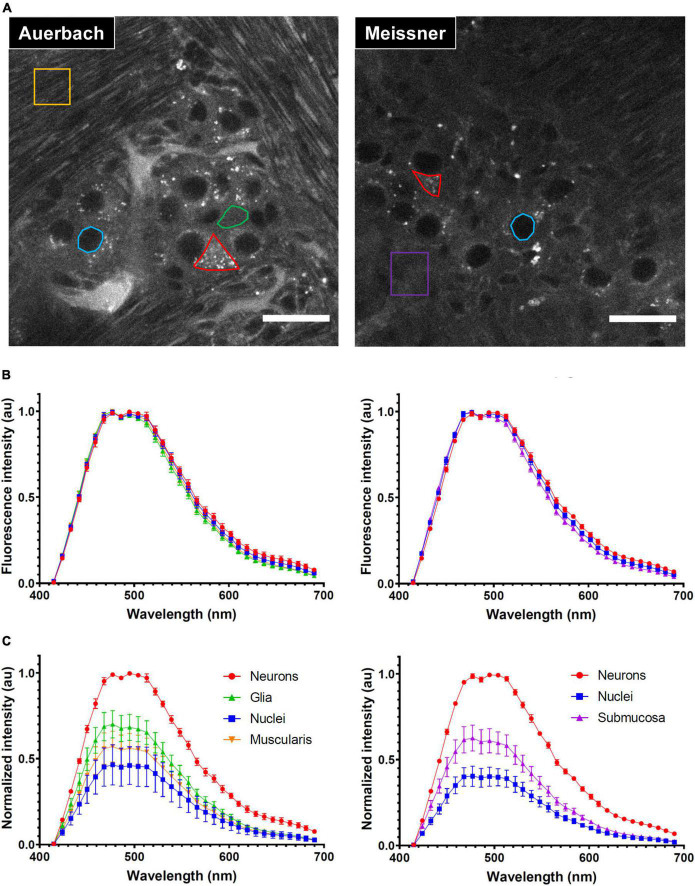
Characterization of the autofluorescence (AF) of the enteric nervous system (ENS). **(A)** Autofluorescence images taken at the level of the Auerbach’s (left) and Meissner’s (right) *plexus*. Representative regions of interest (ROI) used for spectral characterization are shown in color (red: neurons cell bodies, green: enteric glia cytoplasm, blue: neurons nuclei, yellow: *muscularis*, purple: *submucosa*). Scale-bars: 25 μm. **(B)** Spectral signatures of the ROI depicted in panel **(A)** at the level of the Auerbach’s (left) and Meissner’s (right) *plexus*. **(C)** Relative fluorescence intensity of the ROI depicted in panel **(A)** at the level of the Auerbach’s (left) and Meissner’s (right) *plexus*. Error-bars: SD.

Tissue AF is a complex multi-component signal characterized by broad fluorescence excitation and emission spectra. We investigated if spectral information could be used to further refine AF segmentation into different ENS structures. To this end, we selected four ROIs in Auerbach’s plexus: (*i*) neuronal cell bodies that exhibited a strong granular AF (red ROI); (*ii*) cytoplasmic regions of enteric glia (green); (*iii*) neuronal nuclei (blue) and, (*iv*) ROIs within the *muscularis* (yellow) (Figure 4A–left panel). Although we observed no significant spectral differences (Figure 4B–left) we could discriminate different structures based on their intensity that was strongest in neurons, followed by enteric glia, *muscularis* and finally nuclei, which showed the lowest AF and uniform signal. Moreover, neuronal but not glial cells bodies showed numerous brightly AF granules in their cytoplasm (Figure 4C–left).

At the level of Meissner’s plexus, we selected ROIs in 3 structures: neuronal cell bodies (red), neuronal nuclei (blue) and within the *submucosa* (purple) (Figure 4A–right). As previously observed in Auerbach’s plexus, these regions had indistinct AF emission spectra (Figure 4B–right) but they showed similar differences in terms of AF intensity (Figure 4C–*right*).

We conclude that the AF arising from the ENS does not exhibit a sufficient spectral variability upon 405-nm excitation. This is not necessarily a bad thing, because it allows the broad-band detection for maximizing AF collection. The strong intensity contrast observed between different structures of the ENS is sufficient and–together with morphological features–allows for a straightforward identification of cellular and sub-cellular structures.

### 3.5.2-photon spinning-disk microscopy allows fast, deep, and spatially resolved imaging of the non-labeled ENS

We used a modified Nipkow-Petráň-type 2P microscope (Rakotoson et al., 2019) for deep AF imaging. Our instrument produces a 1.5-μm thin, planar non-linear excitation, and with the here used × 25/NA1.1 water objective and detector, we could acquire a 150 × 150 μm diffraction-limited AF image in 480 ms. With a spatial resolution close to the one obtained on a confocal microscope (330 nm laterally) and a temporal resolution at least 10 times faster, our microscope is particularly adapted to studying ENS volumes.

**FIGURE 5:**
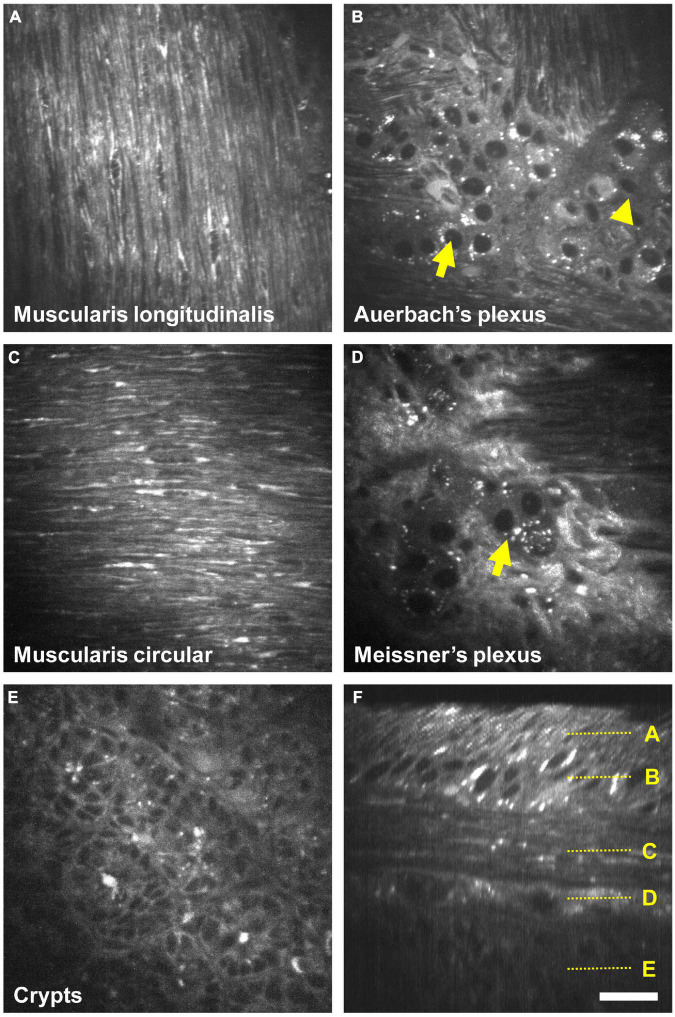
Fast 3D two-photon (2P) imaging of the enteric nervous system (ENS) on the OASIS microscope. **(A–E)** Autofluorescence (AF) images taken upon 800 nm 2P excitation at different depths of the intestinal wall, starting from the *muscularis longitudinalis* down to the crypts (see also Supplementary Videos 1, 2). Arrows: neurons, arrowheads: enteric glia. **(F)** Orthogonal reconstruction based on a *z*-stack acquired with 1 μm steps. Localizations of images **(A–E)** are depicted on the right. Scale-bar: 25 μm.

Figure 5 and Supplementary Videos 1, 2 show AF imaging across the entire cleared intestinal wall, starting from the external face down to the crypts. *Muscularis longitudinalis* (Figure 5A) and *circularis* (Figure 5C) were recognized clearly by their perpendicular arrangement of muscle fibers. In between, Auerbach’s plexus (Figure 5B) exhibited a strong AF contrast and ganglia are readily identified. As observed before upon 1P-excitation, neurons (arrow) but not glia (arrowhead) displayed intense AF granules in their cell bodies upon fs-pulsed 800 nm excitation. Likewise, Meissner’s plexus was characterized by ganglia of systematically fewer neurons compared to Auerbach’s plexus (Figure 5D–arrow). As a consequence of the non-linear excitation, a Second-harmonic Generation (SHG) signal can also be detected in the *submucosa*, arising from fibrous structures. This narrow signal around 400 nm can be separated from the broader AF by appropriate filtering (not shown). Crypts appeared as contrasted structures with sparse AF granules that have earlier be identified as zymogen-rich granules in Paneth cells (Ricard et al., 2012; Figure 5E). The orthogonal (xz) reconstruction (virtual transversal slice) illustrates the cross-sectional reconstruction of the entire intestinal wall, demonstrating the force of label-free imaging in rapidly cleared intestinal tissue (Figure 5F). Of note, using the OASIS 2P microscope, we were able to image AF across 140 μm of the entire mouse intestinal wall (1 μm *z*-step, from the *serosa* to the crypts) in less than 90 s, compared to 15 min–60-fold longer–than on a confocal laser scanning microscope (see Supplementary Video 3 for a direct comparison).

All the work was carried out exclusively on small intestine. However, our technique performs very well on colon (Supplementary Video 4), which is particularly interesting for the analysis of colon biopsies for clinical research and diagnosis.

## 4. Discussion

In the present study, we combined an innovative, patent-pending clearing method that can be geared to preserve different AF components with the unique performance of our single spinning-disk 2P-microscope to demonstrate fast volumetric label-free imaging of the ENS. Our main findings are, (1) label-free imaging permits the unequivocal identification of both Auerbach’s and Meissner’s plexus, validated through immunofluorescence controls on small intestine sections; (2) AF imaging upon 405-nm (1P) or 800-nm (2P) excitation allowed us to unambiguously identify neuronal and glial cell types; (3) the combination of clearing, AF detection and 3-D imaging of *ileum and colon* samples allowed us to reconstruct nervous plexuses in 3-D at a spatial resolution comparable to confocal micrographs (330 nm laterally, 1.5 μm axially) but benefiting from the high throughput (two images/s) of our OASIS 2P microscope. Our approach is of clear interest for morphological studies of a sparse and inhomogeneous structure like the ENS and the label-free imaging allows for a surprisingly detailed analysis of the different layers and cell-types of the enteric system. Our study also underpins the robustness of intrinsic biological signals for 3-D imaging.

Of course, our technique does not replace existing techniques: indeed, ENS is mainly studied using conventional histological slices that are stained or immunolabeled. However, such approach is not optimal for studying a mesh-like and sparse structure like the ENS and different solutions have been proposed to overcome this problem. Among them, the whole-mount technique (Lebouvier et al., 2010a) comprises a careful dissection of the intestinal wall to isolate parts of the ENS that can be immunolabeled and observed. This approach allows for the study of a few ganglia and their connections but it remains relatively time-consuming, requires a strong expertise and it is not free of artifacts. Furthermore, whole-mount preparations isolate the ENS from other structures, the visualization of the interactions and spatial relationships in between different components of the intestinal wall is thus impossible. Once extracted from the surrounding tissue that support its structure, the ENS 3-D architecture is modified. As an alternative, *in vivo* imaging experiments were proposed, but such approaches require the use of transgenic animals that undergo complex surgery and imaging protocols (Kolesnikov et al., 2015; Rakhilin et al., 2016; Motegi et al., 2020) and they are not extensible to human tissue. While *in vivo* recordings can be particularly suitable for physiological experiments (Cirillo et al., 2013; Boesmans et al., 2019; Fung et al., 2021) they may be less adapted for studying the 3-D anatomy of the ENS and its pathologica alterations at a large scale and with a sub-cellular spatial resolution in a short amount of time. Thus, the development of a complete workflow for studying the 3-D architecture of the ENS in its native environment remains of particular interest. We believe that the technique proposed in the present manuscript responds to that need. Our technique is based on, (1) a fast, harmless and easy single step to render the whole intestinal wall transparent, (2) a label-free approach based on tissue AF and SHG detection and, (3) a fast innovative 2P microscope.

We have developed a new ultra-rapid, harmless and efficient clearing method based on a high-RI aqueous solution (Delhomme et al., in preparation). Our approach, named BRG5 will be described once the patent is published. We here demonstrated its use and ease for clearing whole intestinal samples, both *ileum* and colon. Notably, we showed that the plateau of transparency (around 75% quantified in terms of visibility of a stripe pattern) was reached in less than 15 min. The level of transparency reached with our clearing strategy is comparable or higher to what is attained by other techniques. The intestinal sample is transparent enough to allow imaging across its entire wall (from the *serosa* to the crypts) even on a conventional laser-scanning confocal microscope with the pinhole closed down to 1 Airy Unit. Some clearing approaches can generate a higher transparency, however, they require much more time and they are often hazardous for the operator or corrosive to the microscope objective, excluding the use of non-capped high-NA dipping lenses with superior performance as the one used in the present study. Also, many clearing protocols require a depigmentation step before RI matching. In the case of a mildly pigmented organ like the small intestine we found that this step is dispensable with our BRG technique (the colon is almost transparent in the first place). We observed no significant differences in the attained transparency after 15 min clearing, depigmented or not. The slight gain in speed is largely outweighed by the extra 45 min depigmentation step. Moreover, we have also observed a decrease of the AF signal after depigmentation. Avoiding depigmentation, thus not only saves time in the protocol but it preserves AF intensity.

Autofluorescence arises from various biomolecules (elastin, lipofuscin, …) (Zipfel et al., 2003) and it is often a source of unwanted background in immunolabeling experiments, which is why various reagents have been developed for AF removal. Other studies have highlighted the potential of this intrinsic signal for obtaining contrast and perform label-free imaging in various organs and tissues, including the gut (Ricard et al., 2012). Despite its low quantum yield and broad spectral signature, the broad emission allows for an efficient collection. AF detection of the gut was reported upon mono- (Bhattacharjee et al., 2018) and 2-photon excitation (Ricard et al., 2012) in healthy conditions but also for studying pathologies like Hirschsprung’s disease (Aggarwal et al., 2013). In the present study, we employed AF detection for 3-D imaging of the ENS with sub-cellular resolution and we demonstrated that the AF contrast obtained in our images is not based on spectral but intensity differences of the various structures of the intestinal wall. This feature, together with the *a priori* knowledge of the morphology of enteric ganglia can potentially be used for an artificial-intelligence (AI) based segmentation (Ogawa et al., 2021) that we will investigate in future studies. Even without this, our AF images show that ENS components including Auerbach’s and Meissner’s ganglia, the connections in between them, but also neurons and enteric glia within can reliably be discriminated based solely on AF. We confirmed our cell-type identification by immunolabeling against HuC/D, a pan-neuronal marker commonly used to study the ENS (Mazzoni et al., 2021), and against S100β, a more reliable marker for enteric glia than GFAP (Grundmann et al., 2019). Interestingly, the intensities of AF and immunolabeling were commensurable so that both signals could be acquired on the same dual-color (split) image. Thus, the AF signal can be used to enrich immunofluorescence and to provide additional morphological context. This dual approach will increase the information content extracted from a fluorescence image, which can be of particular interest when studying the precise localization of tiny structures in the complex 3D environment of the intestinal wall, or when identifying specific sub-types of cellular populations. A “counterstain” approach is commonly used in pathology labs using conventional histological stains and an immunolabeling protocol revealed by an enzymatic color reaction. But, once again, this approach can only be used on tissue slices (2-D structures) whereas ours works on intact 3-D structures. Of note, standard immunolabeling is also compatible with our BRG clearing protocol, thus enabling observations of labeled and unlabeled structures also in thick samples. This was demonstrated by the HuC/D immunolabeling of neurons of both Auerbach’s and Meissner plexus located at different depths in the intestinal wall. AF can be obtained under mono- and 2-photon excitation. Using the latter approach, SHG imaging of intestinal samples was shown (Ricard et al., 2012). SHG is a non-linear scattering process and enables the acquisition of a signal arising from non-centrosymmetric molecules such as Type-I and -III collagen that can be found in connective tissue (Friedl et al., 2007). SHG signal arising from the *submucosa* was observed during our acquisitions (see Figure 5D) and can be of valuable interest for morphological observations, e.g., for highlighting fibrosis in pathological samples (Ranjit et al., 2016). Also, it can be easily identified and separated from AF due to its relatively narrow peak at half the fundamental wavelength (not shown).

Confocal laser-scanning microscopes are available on many imaging platforms and core facilities, but they are not designed for deep-tissue imaging. While confocal microscopy can be used for 3-D AF imaging of the ENS, the long acquisition times (more than 6 s for a single AF image plane in Figure 3) and volume photobleaching are a clear drawback. Thus, CLSM is suitable for occasional 3-D acquisitions, but it is not recommended for routine imaging of important volumes, large cohorts or for the fast 3-D examination of intestinal samples in pathology labs.

We here demonstrated fast clearing and label-free imaging give very comparable results using different microscopes, including a conventional laser-scanning confocal microscope and a prototype of a single-spinning disk 2-photon microscope (Rakotoson et al., 2019). This planar excitation 2-photon microscope based on a single spinning-disk now allows for considerably faster acquisition times (480 ms/image). Our microscope was successfully tested for 3-D imaging of brain organoids (Rakotoson et al., 2019) and we confirmed its advantages for 3-D AF imaging of the ENS in the present manuscript. Notably, we imaged across the entire mouse intestinal wall (140 μm with a 1 μm *z*-step, from the *serosa* to the crypts) in less than 90 s, compared to 15 min for a CLSM (see Supplementary Video 3). Such speed gains allow for high-throughput studies of the ENS on larger volumes or in numerous samples. One could argue that such characteristics can also be obtained on light-sheet microscopes. However, despite close performances in term of temporal resolution, spatial resolution is lower in comparison to our 2P-spinning disc microscope. Moreover, the geometry of the optical setup in light-sheet microscopes requires the embedding and positioning of the sample for the orthogonal illumination/detection which is not the case for the our 2P spinning-disc microscope that maintains the classical in-line geometry and allows for conventional sample handling.

Using a combination of three approaches that we each optimized independently: clearing, AF detection and fast, volume 2P microscopy, we demonstrated fast, 3D imaging of the fine structure of the ENS that cannot be obtained similarly fast and similarly deep with conventional approaches. AF detection allowed the discrimination of various cellular and subcellular structures including neuronal bodies, nuclei and granule-like structures. Morphological differences in between the Auerbach’s and Meissner’s plexus were highlighted and the connections in between ganglia could be tracked in these label-free preparations. Our approach avoids artifacts due to the slicing process or dissection of the intestinal wall during whole-mount preparations. While this proof-of-concept study was mainly carried out on small intestine samples, it works on colon (see Supplementary Video 4), and we currently use it for human biopsies (data not shown). Applications of our technique are numerous, and include fundamental research on the organization of the ENS and its relationship with its environment and with the central nervous system, as well as pathology, where it can potentially be used for rapid diagnostic purposes in clinical applications. For example, it might contribute to better understand the link in between central aspects of neurodegenerative diseases such as PD and pathologic changes observed at early stages already ENS (Lebouvier et al., 2010b; Ohlsson and Englund, 2019). In either case, our technique allows for high-throughput 3-D imaging and will permit to base such studies on a statistically relevant number of samples. We hope that its ease of its implementation, together with the speed of both clearing and imaging steps will make our technique amenable to a diagnostic use in a hospital environment, potentially even in the operation theater.

## Data availability statement

The raw data supporting the conclusions of this article will be made available by the authors, without undue reservation.

## Ethics statement

This animal study was reviewed and approved by Comité d’éthique en expérimentation animale Buffon CEEA-040.

## Author contributions

DH, CR, and MO designed the experiments, interpreted the results, and wrote the manuscript. DH, BD, and CR performed the experiments. All authors contributed to the article and approved the submitted version.

## Supplementary material

Supplementary Video 1 Fast 3D imaging of the ENS on the OASIS microscope. Autofluorescence xy images taken upon 800 nm excitation at different imaging depths in the intestinal wall starting from the *muscularis longitudinalis* to the crypts. *z*-step: 1 μm; 480 ms/image plane. Orthogonal reconstructions (*yz* and *xz*) are displayed on the right side and below the xy stack, respectively.

Supplementary Video 2 3D rendering and animation of the fine architecture of the ENS based on a z-stack acquired on the OASIS 2P microscope. 800 nm excitation; Z-step: 1 μm; 480 ms/image.

Supplementary Video 3 Comparison of 3D image acquisition on confocal and 2P OASIS microscopes. Autofluorescence xy images taken upon 405 nm excitation (confocal) and taken upon 800 nm excitation (OASIS) at different imaging depths in the intestinal wall starting from the muscularis longitudinalis to the crypts.

Supplementary Video 4 Confocal imaging of the ENS on colon’s thick sample. Autofluorescence xy images taken upon 405 nm excitation at different imaging depths in the colonic wall starting from the *muscularis longitudinalis* to the crypts. *z*-step: 0.91 μm.

## References

[B1] Aggarwal A. Jain M. Frykman P. K. Xu C. Mukherjee S. Muensterer O. J. (2013). Multiphoton microscopy to identify and characterize the transition zone in a mouse model of Hirschsprung disease. *J. Pediatr. Surg.* 48 1288–1293. 10.1016/j.jpedsurg.2013.03.025 23845620 PMC4372128

[B2] Avetisyan M. Schill E. M. Heuckeroth R. O. (2015). Building a second brain in the bowel. *J. Clin. Invest.* 125 899–907. 10.1172/JCI76307 25664848 PMC4362233

[B3] Avilov S. V. (2021). Navigating across multi-dimensional space of tissue clearing parameters. *Methods Appl. Fluoresc.* 9:022001. 10.1088/2050-6120/abe6fb 33592593

[B4] Bewersdorf J. Egner A. Hell S. W. (2006). “Multifocal multi-photon microscopy,” in *Handbook of Biological Confocal Microscopy*, ed. Pawley J. B. (Boston, MA: Springer US), 550–560. 10.1007/978-0-387-45524-2_29

[B5] Bhattacharjee S. Satwaha S. Thornton K. Scholz D. (2018). Label-Free Imaging and Optical Characterization of Tissues Based on Autofluorescence. *ACS Omega* 3 5926–5930. 10.1021/acsomega.8b00678 30023932 PMC6044981

[B6] Bialkowska A. B. Ghaleb A. M. Nandan M. O. Yang V. W. (2016). Improved swiss-rolling technique for intestinal tissue preparation for immunohistochemical and immunofluorescent analyses. *J. Vis. Exp.* 131:54161. 10.3791/54161 27501188 PMC4993444

[B7] Boesmans W. Hao M. M. Fung C. Li Z. Van den Haute C. Tack J. (2019). Structurally defined signaling in neuro-glia units in the enteric nervous system. *Glia* 67 1167–1178. 10.1002/glia.23596 30730592 PMC6593736

[B8] Boesmans W. Martens M. A. Weltens N. Hao M. M. Tack J. Cirillo C. (2013). Imaging neuron-glia interactions in the enteric nervous system. *Front. Cell Neurosci.* 7:183. 10.3389/fncel.2013.00183 24155689 PMC3801083

[B9] Bossolani G. D. P. Pintelon I. Detrez J. D. Buckinx R. Thys S. Zanoni J. N. (2019). Comparative analysis reveals Ce3D as optimal clearing method for in toto imaging of the mouse intestine. *Neurogastroenterol. Motil.* 31:e13560. 10.1111/nmo.13560 30761698

[B10] Cirillo C. Tack J. Vanden Berghe P. (2013). Nerve activity recordings in routine human intestinal biopsies. *Gut* 62 227–235. 10.1136/gutjnl-2011-301777 22387530

[B11] Costa M. Brookes S. J. Hennig G. W. (2000). Anatomy and physiology of the enteric nervous system. *Gut* 47(Suppl. 4), iv15–iv19. 10.1136/gut.47.suppl_4.iv15 11076898 PMC1766806

[B12] Delhomme B. Rakotoson I. Derrien D. Gruere O. Oheim M. (in preparation). *Universal, ultra-fast, and non-toxic tissue clearing for biological fluorescence, biomedical and histological imaging*. Patent FR221109 filed on the 26 Oct 2022.

[B13] Denk W. Strickler J. H. Webb W. W. (1990). Two-photon laser scanning fluorescence microscopy. *Science* 248 73–76. 10.1126/science.2321027 2321027

[B14] Denk W. Svoboda K. (1997). Photon upmanship: why multiphoton imaging is more than a gimmick. *Neuron* 18 351–357. 10.1016/s0896-6273(00)81237-4 9115730

[B15] Egner A. Hell S. W. (2000). Time multiplexing and parallelization in multifocal multiphoton microscopy. *J. Opt. Soc. Am. A Opt. Image Sci. Vis.* 17 1192–1201. 10.1364/josaa.17.001192 10883971

[B16] Friedl P. Wolf K. von Andrian U. H. Harms G. (2007). Biological second and third harmonic generation microscopy. *Curr. Protoc. Cell Biol.* Chapter 4:Unit4.15. 10.1002/0471143030.cb0415s34 18228516

[B17] Fung C. Cools B. Malagola S. Martens T. Tack J. Kazwiny Y. (2021). Luminal short-chain fatty acids and 5-HT acutely activate myenteric neurons in the mouse proximal colon. *Neurogastroenterol. Motil.* 33:e14186. 10.1111/nmo.14186 34121274

[B18] Gershon M. D. (1998). V. Genes, lineages, and tissue interactions in the development of the enteric nervous system. *Am. J. Physiol.* 275 G869–G873. 10.1152/ajpgi.1998.275.5.G869 9815012

[B19] Graham K. D. López S. H. Sengupta R. Shenoy A. Schneider S. Wright C. M. (2020). Robust, 3-dimensional visualization of human colon enteric nervous system without tissue sectioning. *Gastroenterology* 158 2221–2235.e5. 10.1053/j.gastro.2020.02.035 32113825 PMC7392351

[B20] Grundmann D. Loris E. Maas-Omlor S. Huang W. Scheller A. Kirchhoff F. (2019). Enteric Glia: S100, GFAP, and Beyond. *Anat. Rec.* 302 1333–1344. 10.1002/ar.24128 30951262

[B21] Heiss C. N. Olofsson L. E. (2019). The role of the gut microbiota in development, function and disorders of the central nervous system and the enteric nervous system. *J. Neuroendocrinol.* 31:e12684. 10.1111/jne.12684 30614568

[B22] Huisken J. Swoger J. Del Bene F. Wittbrodt J. Stelzer E. H. K. (2004). Optical sectioning deep inside live embryos by selective plane illumination microscopy. *Science* 305 1007–1009. 10.1126/science.1100035 15310904

[B23] Israelyan N. Margolis K. G. (2018). Serotonin as a link between the gut-brain-microbiome axis in autism spectrum disorders. *Pharmacol. Res.* 132 1–6. 10.1016/j.phrs.2018.03.020 29614380 PMC6368356

[B24] Kim K. H. Buehler C. So P. T. (1999). High-speed, two-photon scanning microscope. *Appl. Opt.* 38 6004–6009. 10.1364/ao.38.006004 18324120

[B25] Kobayashi M. Fujita K. Kaneko T. Takamatsu T. Nakamura O. Kawata S. (2002). Second-harmonic-generation microscope with a microlens array scanner. *Opt. Lett.* 27 1324–1326. 10.1364/ol.27.001324 18026438

[B26] Kolesnikov M. Farache J. Shakhar G. (2015). Intravital two-photon imaging of the gastrointestinal tract. *J. Immunol. Methods* 421 73–80. 10.1016/j.jim.2015.03.008 25801674

[B27] Kulkarni S. Ganz J. Bayrer J. Becker L. Bogunovic M. Rao M. (2018). Advances in enteric neurobiology: the “Brain” in the gut in health and disease. *J. Neurosci.* 38 9346–9354. 10.1523/JNEUROSCI.1663-18.2018 30381426 PMC6209840

[B28] Lebouvier T. Coron E. Chaumette T. Paillusson S. Bruley des Varannes S. Neunlist M. (2010a). Routine colonic biopsies as a new tool to study the enteric nervous system in living patients. *Neurogastroenterol. Motil.* 22 e11–e14. 10.1111/j.1365-2982.2009.01368.x 19650774

[B29] Lebouvier T. Neunlist M. Bruley des Varannes S. Coron E. Drouard A. N’Guyen J.-M. (2010b). Colonic biopsies to assess the neuropathology of Parkinson’s disease and its relationship with symptoms. *PLoS One* 5:e12728. 10.1371/journal.pone.0012728 20856865 PMC2939055

[B30] Liu Y.-A. Chen Y. Chiang A.-S. Peng S.-J. Pasricha P. J. Tang S.-C. (2011). Optical clearing improves the imaging depth and signal-to-noise ratio for digital analysis and three-dimensional projection of the human enteric nervous system. *Neurogastroenterol. Motil.* 23 e446–e457. 10.1111/j.1365-2982.2011.01773.x 21895876

[B31] Mahou P. Vermot J. Beaurepaire E. Supatto W. (2014). Multicolor two-photon light-sheet microscopy. *Nat. Methods* 11 600–601. 10.1038/nmeth.2963 24874570

[B32] Mazzoni M. Caremoli F. Cabanillas L. de Los Santos J. Million M. Larauche M. (2021). Quantitative analysis of enteric neurons containing choline acetyltransferase and nitric oxide synthase immunoreactivities in the submucosal and myenteric plexuses of the porcine colon. *Cell Tissue Res.* 383 645–654. 10.1007/s00441-020-03286-7 32965550 PMC8059758

[B33] Motegi Y. Sato M. Horiguchi K. Ohkura M. Gengyo-Ando K. Ikegaya Y. (2020). Confocal and multiphoton calcium imaging of the enteric nervous system in anesthetized mice. *Neurosci. Res.* 151 53–60. 10.1016/j.neures.2019.02.004 30790590

[B34] Neckel P. H. Mattheus U. Hirt B. Just L. Mack A. F. (2016). Large-scale tissue clearing (PACT): Technical evaluation and new perspectives in immunofluorescence, histology, and ultrastructure. *Sci. Rep.* 6:34331. 10.1038/srep34331 27680942 PMC5041186

[B35] Nielsen T. Fricke M. Hellweg D. Andresen P. (2001). High efficiency beam splitter for multifocal multiphoton microscopy. *J. Microsc.* 201 368–376. 10.1046/j.1365-2818.2001.00852.x 11240852

[B36] Ogawa K. Oshima Y. Etoh T. Kaisyakuji Y. Tojigamori M. Ohno Y. (2021). Label-free detection of human enteric nerve system using Raman spectroscopy: A pilot study for diagnosis of Hirschsprung disease. *J. Pediatr. Surg.* 56 1150–1156. 10.1016/j.jpedsurg.2021.03.040 33838894

[B37] Oheim M. Beaurepaire E. Chaigneau E. Mertz J. Charpak S. (2001). Two-photon microscopy in brain tissue: parameters influencing the imaging depth. *J. Neurosci. Methods* 111 29–37. 10.1016/s0165-0270(01)00438-1 11574117

[B38] Ohlsson B. Englund E. (2019). Atrophic Myenteric and Submucosal Neurons Are Observed in Parkinson’s Disease. *Parkinsons. Dis.* 2019:7935820. 10.1155/2019/7935820 31321021 PMC6607708

[B39] Orzekowsky-Schroeder R. Klinger A. Martensen B. Blessenohl M. Gebert A. Vogel A. (2011). In vivo spectral imaging of different cell types in the small intestine by two-photon excited autofluorescence. *J. Biomed. Opt.* 16:116025. 10.1117/1.3655587 22112130

[B40] Rakhilin N. Barth B. Choi J. Muñoz N. L. Kulkarni S. Jones J. S. (2016). Simultaneous optical and electrical in vivo analysis of the enteric nervous system. *Nat. Commun.* 7:11800. 10.1038/ncomms11800 27270085 PMC4899629

[B41] Rakotoson I. Delhomme B. Djian P. Deeg A. Brunstein M. Seebacher C. (2019). Fast 3-D imaging of brain organoids with a new single-objective planar-illumination two-photon microscope. *Front. Neuroanat.* 13:77. 10.3389/fnana.2019.00077 31481880 PMC6710410

[B42] Ranjit S. Dobrinskikh E. Montford J. Dvornikov A. Lehman A. Orlicky D. J. (2016). Label-free fluorescence lifetime and second harmonic generation imaging microscopy improves quantification of experimental renal fibrosis. *Kidney Int.* 90 1123–1128. 10.1016/j.kint.2016.06.030 27555119 PMC5473685

[B43] Ricard C. Arroyo E. D. He C. X. Portera-Cailliau C. Lepousez G. Canepari M. (2018). Two-photon probes for in vivo multicolor microscopy of the structure and signals of brain cells. *Brain Struct. Funct.* 223 3011–3043. 10.1007/s00429-018-1678-1 29748872 PMC6119111

[B44] Ricard C. Vacca B. Weber P. (2012). Three-dimensional imaging of small intestine morphology using non-linear optical microscopy and endogenous signals. *J. Anat.* 221 279–283. 10.1111/j.1469-7580.2012.01529.x 22697278 PMC3458633

[B45] Richardson D. S. Guan W. Matsumoto K. Pan C. Chung K. Ertürk A. (2021). Tissue clearing. *Nat. Rev. Methods Primers* 1:84. 10.1038/s43586-021-00080-9 35128463 PMC8815095

[B46] Richardson D. S. Lichtman J. W. (2015). Clarifying tissue clearing. *Cell* 162 246–257. 10.1016/j.cell.2015.06.067 26186186 PMC4537058

[B47] Rogart J. N. Nagata J. Loeser C. S. Roorda R. D. Aslanian H. Robert M. E. (2008). Multiphoton imaging can be used for microscopic examination of intact human gastrointestinal mucosa ex vivo. *Clin. Gastroenterol. Hepatol.* 6 95–101. 10.1016/j.cgh.2007.10.008 18065276 PMC2254558

[B48] Schneider S. Wright C. M. Heuckeroth R. O. (2019). Unexpected roles for the second brain: enteric nervous system as master regulator of bowel function. *Annu. Rev. Physiol.* 81 235–259. 10.1146/annurev-physiol-021317-121515 30379617

[B49] Schindelin J. Arganda-Carreras I. Frise E. Kaynig V. Longair M. Pietzsch T. (2012). Fiji: An open-source platform for biological-image analysis. *Nat. Methods* 9, 676–682. 10.1038/nmeth.2019 22743772 PMC3855844

[B50] Shimozawa T. Yamagata K. Kondo T. Hayashi S. Shitamukai A. Konno D. (2013). Improving spinning disk confocal microscopy by preventing pinhole cross-talk for intravital imaging. *Proc. Natl. Acad. Sci. U.S.A.* 110 3399–3404. 10.1073/pnas.1216696110 23401517 PMC3587224

[B51] Straub M. Lodemann P. Holroyd P. Jahn R. Hell S. W. (2000). Live cell imaging by multifocal multiphoton microscopy. *Eur. J. Cell Biol.* 79 726–734. 10.1078/0171-9335-00105 11089921

[B52] Tian T. Yang Z. Li X. (2021). Tissue clearing technique: Recent progress and biomedical applications. *J. Anat.* 238 489–507. 10.1111/joa.13309 32939792 PMC7812135

[B53] Truong T. V. Supatto W. Koos D. S. Choi J. M. Fraser S. E. (2011). Deep and fast live imaging with two-photon scanned light-sheet microscopy. *Nat. Methods* 8 757–760. 10.1038/nmeth.1652 21765409

[B54] Ueda H. R. Ertürk A. Chung K. Gradinaru V. Chédotal A. Tomancak P. (2020). Tissue clearing and its applications in neuroscience. *Nat. Rev. Neurosci.* 21 61–79. 10.1038/s41583-019-0250-1 31896771 PMC8121164

[B55] Weiss K. R. Voigt F. F. Shepherd D. P. Huisken J. (2021). Tutorial: practical considerations for tissue clearing and imaging. *Nat. Protoc.* 16 2732–2748. 10.1038/s41596-021-00502-8 34021294 PMC10542857

[B56] Yuan P.-Q. Bellier J.-P. Li T. Kwaan M. R. Kimura H. Taché Y. (2021). Intrinsic cholinergic innervation in the human sigmoid colon revealed using CLARITY, three-dimensional (3D) imaging, and a novel anti-human peripheral choline acetyltransferase (hpChAT) antiserum. *Neurogastroenterol. Motil.* 33:e14030. 10.1111/nmo.14030 33174295 PMC8126258

[B57] Zipfel W. R. Williams R. M. Christie R. Nikitin A. Y. Hyman B. T. Webb W. W. (2003). Live tissue intrinsic emission microscopy using multiphoton-excited native fluorescence and second harmonic generation. *Proc. Natl. Acad. Sci. U.S.A.* 100 7075–7080. 10.1073/pnas.0832308100 12756303 PMC165832

[B58] Zong W. Zhao J. Chen X. Lin Y. Ren H. Zhang Y. (2015). Large-field high-resolution two-photon digital scanned light-sheet microscopy. *Cell Res.* 25 254–257. 10.1038/cr.2014.124 25257466 PMC4650563

